# Case Report: First Report and Phylogenetic Analysis of Porcine Astroviruses in Chile

**DOI:** 10.3389/fvets.2021.764837

**Published:** 2021-11-25

**Authors:** Carlos Flores, Naomi Ariyama, Benjamín Bennett, Juan Mena, Claudio Verdugo, Sunil Mor, Barbara Brito, Galia Ramírez-Toloza, Victor Neira

**Affiliations:** ^1^Departamento de Medicina Preventiva Animal, Facultad de Ciencias Veterinarias y Pecuarias, Universidad de Chile, La Pintana, Chile; ^2^Instituto de Patología Animal, Universidad Austral de Chile, Valdivia, Chile; ^3^College of Veterinary Medicine, University of Minnesota, Saint Paul, MN, United States; ^4^The Ithree Institute, University of Technology Sydney, Sydney, NSW, Australia

**Keywords:** porcine astrovirus, poAstV, swine intensive farm, next-generating sequencing, phylogenetic analysis

## Abstract

Porcine Astrovirus (PoAstV) causes mild diarrhea in young pigs and is considered an emerging virus in the swine industry worldwide. PoAstV has high genetic diversity and has been classified into five genetic lineages, PoAstV1–5. In Chile, only human astroviruses have been reported. This study aimed to determine the presence and genetic diversity of PoAstV circulating in intensive pig farms in Chile. Seventeen Chilean intensive swine farms from Valparaíso, Metropolitana, O'Higgins, Ñuble and Araucanía regions were sampled. A selection of oral fluid and fecal material samples from 1–80 days-old pigs were collected and analyzed using next-generation sequencing. The circulation of PoAstV was confirmed in all studied farms. We obtained complete or partial sequences of PoAstV-2 (*n* = 3), PoAstV-4 (*n* = 2), and PoAstV-5 (*n* = 7). In 15 out of 17 farms, we detected more than one lineage co-circulating. Phylogenetic analyses grouped the seven PoAstV-5 strains in a monophyletic cluster, closely related to the United States PoAstV-5 strains. The three PoAstV-2 were located into two separate sub-clusters. PoAstV-4 sequences are also grouped in two different clusters, all related to Japanese strains. Thus, our results indicate that PoAstV circulates in Chile with high frequency and diversity. However, the lack of reference sequences impairs local evolution patterns establishment and regional comparisons. This is the first contribution of PoAstV genomes in Latin America; more studies are needed to understand the diversity and impact of PoAstV on swine health.

## Introduction

Astroviruses (AstVs) are emerging pathogens, belonging to members of the family Astroviridae. These viruses are divided into two genera: *Mamastrovirus* and *Avastrovirus*, which infect mammals and birds, respectively (1). AstV infections cause a wide range of clinical signs from gastroenteric (e.g., human, turkey, sheep, and pig) to neurologic (e.g., human, mink, cattle, sheep, and pig) disease (2–10). They are non-enveloped small viruses (30 nm) with a positive-sense single-stranded RNA genome of 6.4–7.9 kb (11). The genome contains three open reading frames (ORFs): the ORF1a and ORF1b, encoding non-structural proteins, and the ORF2, which encodes the capsid (11, 12).

In humans, AstV is the third most common cause of viral diarrhea in young children worldwide, with high seroprevalences as 94% in children of 6–9 years old (13, 14). Despite this high prevalence, due to the lack of cell culture systems and animal models, AstV are among the least studied enteric RNA viruses (12). However, advances in sequencing technologies have increased the availability of genome sequences and the identification of new strains. Typically, human AstV (HAstV) infections cause acute self-limiting mild diarrhea (12). Nevertheless, immunocompromised patients occasionally exhibit systemic spread, resulting in neurologic disease (15). In addition, a zoonotic potential of AstV is suspected but remains unclear (16–18). Genetic and evolutionary studies support the idea that both cross-species transmission and recombination events among AstV of human, porcine, and other species origin, may have occurred (11, 16).

Porcine astrovirus (PoAstV) has high genetic diversity, it is worldwide distributed, and it is commonly detected and shed by healthy and diarrheic swine (19). Five lineages of porcine AstV (PoAstV1–5) have been described by Laurin, Dastor (20), but only PoAstV-1 has been officially classified under the species *Mamastrovirus 3* (21). In the US, pigs are commonly (13.9%) co-infected with multiple astrovirus strains (19). High prevalence and co-infections may create appropriate conditions for viral recombination and the potential emergence of viral variants that pose a higher risk of clinical disease. Recently, PoAstV has been linked to extraintestinal infections suggesting more complex pathogenesis and serious outcomes than previously thought (3, 4, 22).

In Chile, a recent study reported human AstV infections (14%) as a predominant cause of viral gastroenteritis in rural zones, in addition to norovirus (15%) and rotavirus (14%) (23). However, animal AstV has not been described in Chile. Considering the high prevalence and worldwide distribution of the AstV and PoAstV, the suspected zoonotic potential, and the lack of information regarding these viruses in a regional context, this study aims to determine the presence and genetic diversity of PoAstV circulating in Chilean intensive pig farms. These samples were taken in the context of other viral disease surveillance programs.

## Materials and Methods

### Sample Collection

During influenza virus and rotavirus surveillance and diagnosis programs, we collected oral fluids and fecal samples from 1–80 days-old pigs in 17 intensive pig farms from mainland Chile in 2015 and 2017. The sampled farms are located in an area that concentrates 95% of the national intensive pig production (Valparaíso, Metropolitana, Libertador General Bernardo O'Higgins, Maule, Ñuble, and Araucanía Regions), which represents approximately 50% of the pig inventory in Chile (24, 25). Each fecal sample corresponds to a pool of 5 diarrheic feces, which were collected using nylon gloves, deposited in sterile 50 mL tubes with 20 mL of viral transport media (Minimum Essential Medium, 1X Trypsin TPCK, 2% bovine serum albumin, and 1% antifungal antibiotic solution), and then centrifuged at 7,000 rpm for 5 min. Oral fluids were collected by groups of 20–30 healthy pigs kept in pens. Briefly, a 16 mm braided cotton rope was hung in each pen for about 30 min. The ropes were deposited inside plastic ziplock bags and squeezed to obtain the oral fluid and deposited into 50 mL tubes. All samples were kept at −20°C until processing. One sample per farm (*n* = 17) was selected for next-generation sequencing. The criteria to select the samples included the location, geographic distance between farms and detection of other pathogens such as rotavirus and influenza.

### Viral RNA Extraction and Whole-Genome Sequencing

The RNA extraction was carried out using the Chomczynski-phenol solution (Winkler, BM-1,755, Chile) following the manufacturer's recommendations. The Next-generation sequencing (NGS) was performed at the Molecular Diagnostic Development Laboratory at the Veterinary Diagnostic Laboratory of the University of Minnesota (MVDL, UMN), USA, using the Illumina MiSeq platform. Library pre-paration was performed using the SMARTer Stranded Total RNA-Seq Kit v2–Pico Input Mammalian (Takara bio, USA). *De novo* assembly of the reads was carried out using an automated pipeline that identifies viral reads using DIAMOND protein alignment and the Swissprot Uniref90 database. The viral reads are then grouped by the lowest common ancestor and assembled using SPAdes and subsequently, the contigs are joined using an Advanced Genome Aligner (http://www.genomedetective.com/app/typingtool/virus/). Complementary, the assembly using PoAstV reference sequences was performed using Geneious Prime® 2021.2.2.

### Phylogeny

Complete or near to complete Chilean PoAstV genomes, with >77% of coverage, were used for phylogenetic analysis (Table 1). These sequences were compared with all complete or near complete PoAstV genome available in GenBank database. We used the lineage classification described by Lee et al. (26). The final data set comprised 93 Astrovirus genome sequences that were aligned using MUSCLE (27). The phylogeny was constructed using RAxML with the GTR+G+I substitution model and 1,000 bootstrap replications in Geneious Prime® 2021.2.2. Additionally, the phylogeny was constructed for the ORF2 region with the same methodology, and p-distances at the nucleotide and amino acid level of the ORF2 sequences were estimated using MEGA X (28).

**Table 1:** Summary of porcine astroviruses whole-genome sequencing results obtained from intensive farms in Chile.

**Farm**	**Region**	**Sample**	**Strains**	**Accession number**	**Sequencing**					**ORF1a**		**ORF1b**		**ORF2**	
					**Coverage (%)**	**Contigs**	**Astrovirus reads**	**Reads total**	**Astrovirus reads rate (%)**	**Length**	**AAC**	**Length**	**AAC**	**Length**	**AAC**
1	V	Feces	PoAstV-5/Swine/CHI/ FB016/2017	MZ819168	100	1	187,438	259,201	72.3	2,634	877	1,452	483	2,346	781
1	V	Feces	PoAstV-4/Swine/CHI/ FB016/2017	MZ819174	87	2	19,482	259,201	7.5	2,622	873	NA	NA	2,448	815
2	RM	Feces	PoAstV-5/Swine/CHI/ FB036/2017	MZ819170	100	1	35,538	76,494	46.5	2,619	872	1,452	483	2,346	781
2	RM	Feces	PoAstV-4/Swine/CHI/ FB036/2017	MZ819173	77	1	2,828	76,494	3.7	2,622	873	1,098	365	2,217	739
2	RM	Feces	PoAstV-2/Swine/CHI/ FB036/2017	MZ819164	100	1	5,429	76,494	7.1	2,490	829	1,119	372	2,445	814
3	VI	Oral fluid	PoAstV-5/Swine/CHI/ FB033/2017	MZ819171	100	NA	NA	NA	NA	2,619	872	1,452	483	2,346	781
9	IX	Oral fluid	PoAstV-5/Swine/CHI/ FB0148/2015	MZ819166	96	1	2,531	5,670	44.6	2,619	872	1,452	483	2,241	747
9	IX	Oral fluid	PoAstV-2/Swine/CHI/ FB0148/2015	MZ819163	91	4	2,326	5,670	41	1,143	380	885	294	1,539	512
14	VII	Oral fluid	PoAstV-5/Swine/CHI/ CF2671/2017	MZ819167	99	1	1,003	5,365	18.7	2,619	872	1,452	483	2,346	781
15	VII	Oral fluid	PoAstV-5/Swine/CHI/ CF2672/2017	MZ819169	99	1	1,684	16,506	10.2	1,347	448	1,452	483	2,346	781
16	VII	Oral fluid	PoAstV-2/Swine/CHI/ CF2673/2017	MZ819165	100	1	1,717	15,736	10.9	2,502	833	1,119	372	2,436	811
17	VI	Feces	PoAstV-5/Swine/CHI/ FB032/2017	MZ819172	100	NA	NA	NA	NA	2,619	872	1,452	483	2,346	781

*V, Valparaíso; RM, Metropolitana; VI, Libertador General Bernardo O'Higgins; VII, Maule; IX, Araucanía; NA, Not available*.

## Results and Discussion

All samples were successfully sequenced by Illumina. The most consistent virus family found in the samples was *Astroviridae*. Additionally, reads that belong to other families, such as *Caliciviridae, Parvoviridae*, and *Reoviridae*, were found at lower rates and were not considered for further analysis as they are beyond the aim of this study. Porcine astrovirus reads were observed in all samples/farms included, confirming its ubiquity in Chilean swine intensive production. Overall results of PoAstV identified 229,496 reads for PoAstV-5, 33,917 for PoAstV-4 and 31,751 for PoAstV-2. Only 10 reads were classified as PoAstV3, however, the limited reads do not provide sufficient evidence to confirm the presence of this lineage.

PoAstV-4 was identified in 16 out of 17 farms, PoAstV-2 in 15 and PoAstV-5 in 13 farms (Supplementary Table 1). These results are in agreement with estimations made in the US and some European and Asian countries, where the most prevalent lineage is PoAstV-4, mainly followed by PoAstV-2 (19, 29–33). Contrary to the situation in China, where the most widely distributed strain is presumably PoAstV-2 (34).

We identified at least two different PoAstV lineages co-circulating in 15 out of 17 farms (Supplementary Table 1). Thus, the Chilean swine exhibits conditions for PoAstV recombination events. The circulation of multiple PoAstV strains in the same farm has been reported previously in China, Denmark, Slovakia, Thailand, and the USA (31, 35–37). Co-infection of different lineages in the same individual has also been reported (19, 38).

**Figure 1:**
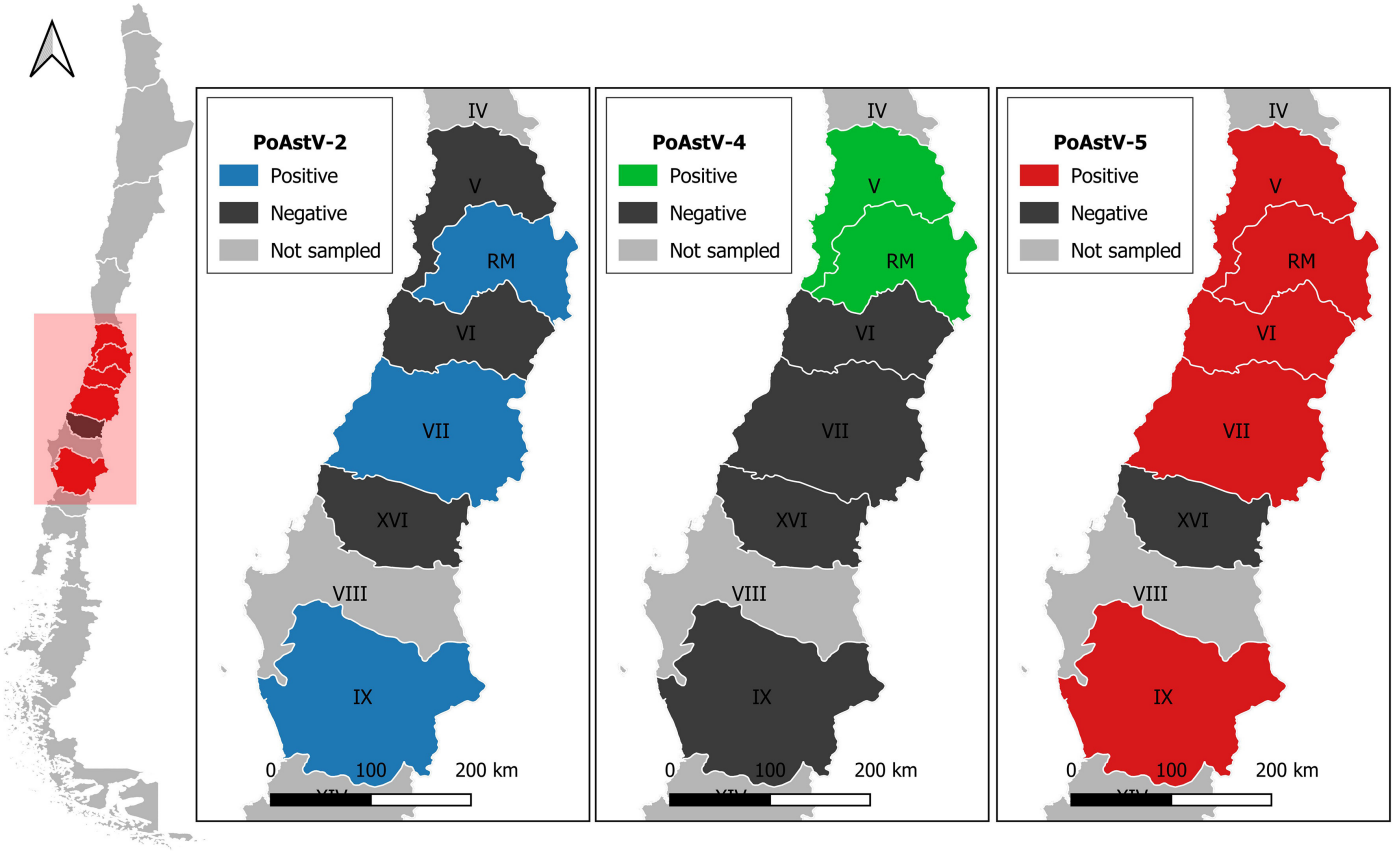
PoAstV strains with >77% of sequencing coverage were detected in Porcine intensive farms from different regions of Chile. Regions are labeled as V, Valparaíso; RM, Metropolitana; VI, Libertador General Bernardo O'Higgins; VII, Maule; XVI, Ñuble; IX, Araucanía.

Eleven complete or near-to-complete genomes of PoAstV were obtained, which were used for the phylogenetic analysis (Table 1). The genomes were obtained from both fecal and oral fluids samples and were recovered from 8 different farms. Figure 1 shows the geographic distribution of the sequences obtained (Figure 1). Interestingly, from farm two, it was possible to obtain the genome of PoAst-2, 4, and 5.

**Figure 2:**
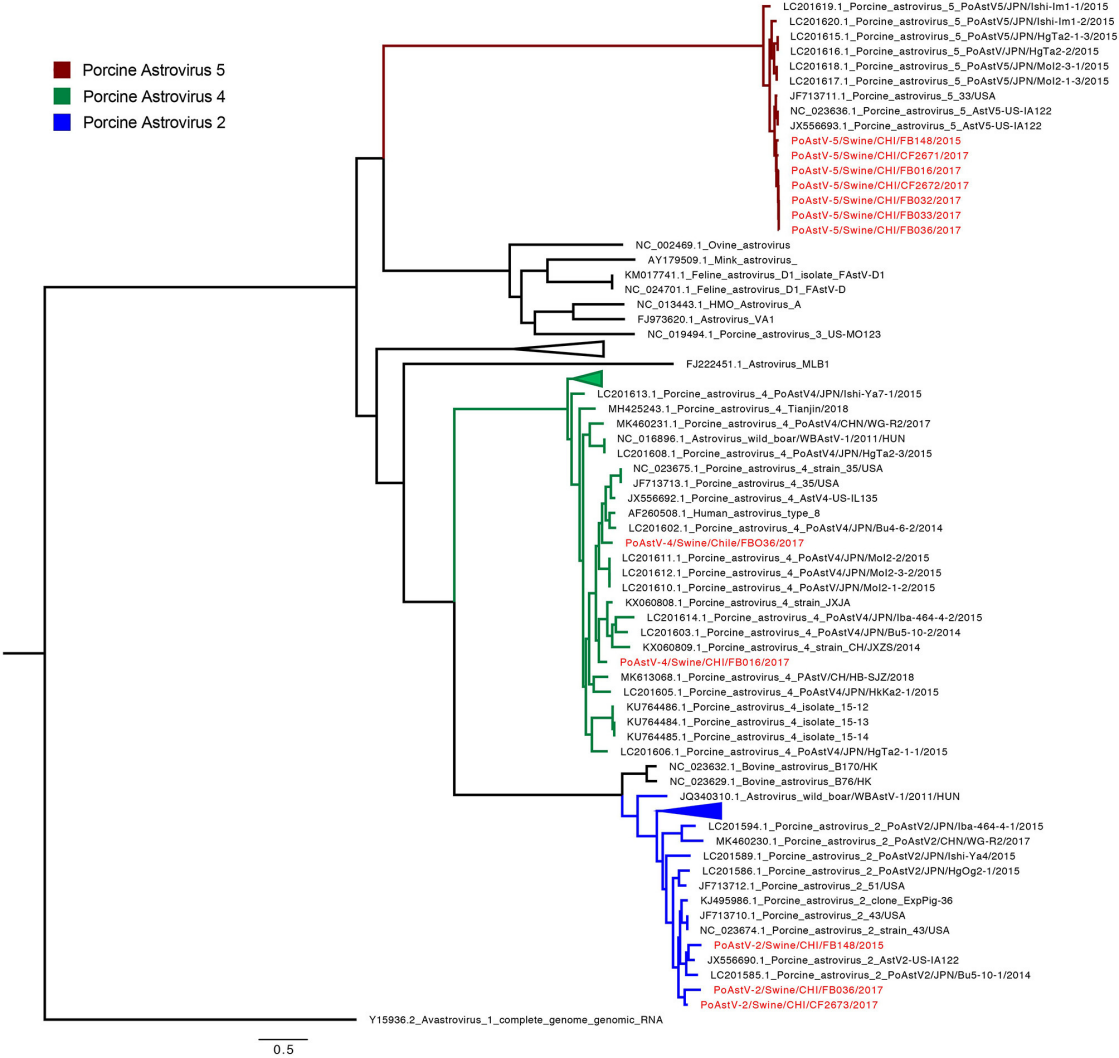
Phylogenetic tree of PoAstV by using the complete genome. The final dataset included 93 genomes. Chilean sequences are highlighted in red. Clusters by species are highlighted in colors: PoAstV-5 (Red), PoAstV-4 (Green) and PoAstV-2 (Blue).

The phylogenetic analysis grouped the Chilean strains into 5 (Figure 2 and Supplementary Figure 1). The seven PoAstV-5 strains are grouped into one monophyletic cluster (97.4% pairwise identity), closely related to strains detected in the USA. Interestingly, the PoAstV-5 were obtained from seven different farms distributed in five geographic regions. The three PoAstV-2 genomes were detected from three different farms in different regions and grouped into two separate sub-clusters. The PoAstV-2/Swine/CHI/FB036/2017 (Metropolitan region) and PoAstV-2/Swine/CHI /CF2673/2017 (Maule region) genomes formed one sub-cluster, while PoAstV-2 /Swine/CHI/FB0148/2017 (Araucania region) grouped with sequences from USA and Japan. Finally, the two PoAstV-4 from two different farms and regions (Metropolitan and Valparaiso region) were phylogenetically distant also related to Japanese strains. The phylogeny demonstrates a genetic relationship between Chilean PoAstV-5 strains, suggesting the same origin for those strains. On the contrary, PoAstV-2 and PoAstV-4 results indicate more diversity even with fewer sequences (For details on genetic distances see Supplementary Tables 2, 3).

Due to the limited number of sequences obtained in this study and the scarce of sequences in GenBank database, conclusions about the origin of the viral strains cannot be elucidated. Indeed, most of the available sequences in GenBank database are from the USA, Japan, and China. Other limitations of the phylogenetic analysis are the incomplete genome coverage in several samples (Table 1) and the potential errors derived from sequencing methods, as these may alter the phylogenetic tree estimation.

This is the first report characterizing the PoAstV sequences circulating in Chile.

This result represents, in turn, the first PoAstV genomes from swine in Latin America. PoAstV studies in Latin America are very scarce, and only two have been published. One study identified PoAstV in healthy pigs from a farm in Brazil (39), and another study conducted in Colombia, which obtained partial PoAstV sequences from diarrheic piglets and humans (40). To date, most of the PoAstV sequences available in GenBank database were obtained in the Northern hemisphere.

Our results support the detection of PoAstV in the Chilean swine population, similar to other observations worldwide. Further studies are needed to understand the relevance of PoAstV to swine health and the evolution and spread of PoAstV locally and globally.

## Data Availability Statement

The datasets presented in this study can be found in online repositories. The names of the repository/repositories and accession number(s) can be found in the article/Supplementary Material.

## Ethics Statement

The animal study was reviewed and approved by Institutional Animal Care and Use Committees of the Universidad de Chile, protocol number 02–2016. Written informed consent was obtained from the owners for the participation of their animals in this study.

## Author Contributions

NA, GR-T, and VN: study design and conceptualization. SM and VN: funding and resources. CF and JM: samples collection and processing. CF, JM, and SM: performed the assays. CF, NA, CV, BB, and VN: data analysis. CF, NA, GR-T, and VN: wrote the paper. All authors critically evaluated the paper. All authors contributed to the article and approved the submitted version.

## Funding

Animal Virology Laboratory, Faculty of Veterinary and Animal Sciences, Universidad de Chile and Programa Fondecyt 11170877 and 1211517.

## Conflict of Interest

The authors declare that the research was conducted in the absence of any commercial or financial relationships that could be construed as a potential conflict of interest.

## Publisher's Note

All claims expressed in this article are solely those of the authors and do not necessarily represent those of their affiliated organizations, or those of the publisher, the editors and the reviewers. Any product that may be evaluated in this article, or claim that may be made by its manufacturer, is not guaranteed or endorsed by the publisher.
