# Supplementary material for: Case Report: First Report and Phylogenetic Analysis of Porcine Astroviruses in Chile
Source: Front Vet Sci. 2021 Nov 25;8:764837. doi: 10.3389/fvets.2021.764837 (PMC8656452; doi:10.3389/fvets.2021.764837)
Supplement: Supplementary file 1 [file Data_Sheet_1.pdf]

Supplementary Material

Supplementary Table 1. Sample identification, method of collection and positivity for the different genotypes of porcine astroviruses (PoAstV) in each analyzed farm in Chile.

Farm	Code	Region	Sample	*PoAstV-2*	*PoAstV-3*[#]	*PoAstV-4*	*PoAstV-5*
1	FBO16	Valparaíso	Feces	X		X	X
2	FBO36	Metropolitana	Feces	X		X	X
3	FBO33	O´Higgins	Oral Fluid	X		X	
4	FBO58	Ñuble	Feces	X	X	X	X
5	FBO46	Ñuble	Feces	X		X	X
6	FBO50	Ñuble	Oral Fluid	X		X	
7	FB062	O´Higgins	Oral Fluid			X	
8	FBO84	O´Higgins	Oral Fluid	X		X	X
9	FBO148	Araucanía	Oral Fluid	X		X	X
10	FBO155	Metropolitana	Oral Fluid	X		X	X
11	FBO66	O´Higgins	Oral Fluid	X		X	X
12	CF2669	Maule	Oral Fluid	X		X	X
13	CF2670	Maule	Oral Fluid	X		X	X
14	CF2671	Maule	Oral Fluid	X		X	X
15	CF2672	Maule	Oral Fluid			X	X
16	CF2673	Maule	Oral Fluid	X		X	X
17	FBO32	O´Higgins	Feces	X			
	Total of positive farms (%)			15 (88)	1(6)	16 (94)	13 (76)

[#]Low number of sequencing reads (n=10)

Supplementary Table 2. Estimates of divergence at nucleotide level between PoAstV ORF2 sequences from Chile and representative PoAstV2, 4, and 5. The number of base differences per site from between sequences are shown. Standard error estimates are shown above the diagonal. There were a total of 3895 positions in the final dataset.

		1	2	3	4	5	6	7	8	9	10	11	12	13	14	15	16	17	18
1	PoAstV-2/Swine/CHI/FB036/2017		0.010	0.009	0.008	0.011	0.011	0.016	0.011	0.011	0.011	0.011	0.011	0.011	0.011	0.011	0.011	0.011	0.011
2	PoAstV-2/Swine/CHI/CF2673/2017	0.334		0.011	0.011	0.010	0.011	0.017	0.011	0.011	0.011	0.011	0.011	0.011	0.011	0.011	0.011	0.011	0.011
3	PoAstV-2/Swine/CHI/FB148/2015	0.205	0.340		0.009	0.012	0.011	0.018	0.012	0.011	0.012	0.012	0.012	0.011	0.011	0.011	0.012	0.012	0.012
4	JF713710.1_Porcine_astrovirus_2_43/USA	0.167	0.332	0.209		0.011	0.011	0.017	0.011	0.011	0.011	0.011	0.011	0.011	0.011	0.011	0.011	0.011	0.011
5	LC201592.1_PoAstV2/JPN/Ishi-Ya8/2015	0.413	0.418	0.415	0.413		0.011	0.017	0.011	0.011	0.011	0.011	0.011	0.011	0.011	0.011	0.011	0.011	0.011
6	PoAstV-4/Swine/CHI/FB016/2017	0.535	0.539	0.529	0.531	0.534		0.014	0.009	0.010	0.011	0.011	0.011	0.011	0.011	0.011	0.011	0.011	0.011
7	PoAstV-4/Swine/Chile/FBO36/2017	0.511	0.489	0.496	0.506	0.516	0.245		0.013	0.014	0.017	0.017	0.017	0.017	0.017	0.017	0.018	0.017	0.017
8	JF713713.1_Porcine_astrovirus_4_35/USA	0.546	0.533	0.536	0.545	0.545	0.307	0.197		0.010	0.011	0.011	0.011	0.011	0.011	0.011	0.011	0.011	0.011
9	LC201600.1_PoAstV4/JPN/Bu4-2-2/2014	0.549	0.534	0.525	0.534	0.540	0.388	0.254	0.387		0.011	0.011	0.011	0.011	0.011	0.011	0.011	0.011	0.011
10	PoAstV-5/Swine/CHI/CF2671/2017	0.588	0.576	0.575	0.581	0.564	0.552	0.542	0.571	0.559		0.004	0.004	0.004	0.004	0.004	0.004	0.005	0.009
11	PoAstV-5/Swine/CHI/CF2672/2017	0.585	0.575	0.577	0.577	0.566	0.553	0.547	0.570	0.550	0.035		0.002	0.002	0.002	0.002	0.004	0.005	0.009
12	PoAstV-5/Swine/CHI/FB016/2017	0.583	0.574	0.574	0.576	0.564	0.553	0.544	0.570	0.551	0.033	0.011		0.002	0.002	0.002	0.004	0.005	0.009
13	PoAstV-5/Swine/CHI/FB032/2017	0.585	0.574	0.575	0.578	0.563	0.556	0.543	0.571	0.552	0.034	0.013	0.013		0.001	0.000	0.004	0.005	0.009
14	PoAstV-5/Swine/CHI/FB033/2017	0.585	0.574	0.575	0.578	0.563	0.556	0.543	0.571	0.552	0.034	0.014	0.014	0.001		0.001	0.004	0.005	0.009
15	PoAstV-5/Swine/CHI/FB036/2017	0.585	0.574	0.575	0.578	0.563	0.556	0.543	0.571	0.552	0.034	0.013	0.013	0.000	0.001		0.004	0.005	0.009
16	PoAstV-5/Swine/CHI/FB148/2015	0.585	0.579	0.579	0.578	0.564	0.557	0.546	0.575	0.562	0.037	0.039	0.037	0.038	0.039	0.038		0.005	0.009
17	JF713711.1_Porcine_astrovirus_5_33/USA	0.579	0.580	0.579	0.580	0.569	0.554	0.543	0.572	0.563	0.068	0.072	0.068	0.069	0.070	0.069	0.071		0.009
18	LC201619.1_PoAstV5/JPN/Ishi-Im1-1/2015	0.581	0.587	0.565	0.569	0.581	0.554	0.548	0.563	0.559	0.211	0.214	0.212	0.216	0.216	0.216	0.215	0.210	

Supplementary Table 3. Estimates of divergence at amino acid level between PoAstV ORF2 sequences from Chile and representative PoAstV2, 4, and 5. The number of amino acid differences per site from between sequences are shown. Standard error estimates are shown above the diagonal. The coding data was translated assuming a Standard genetic code table. There were a total of 1148 positions in the final dataset.

		1	2	3	4	5	6	7	8	9	10	11	12	13	14	15	16	17	18
1	PoAstV-2/Swine/CHI/FB036/2017		0.021	0.022	0.022	0.021	0.017	0.029	0.017	0.018	0.017	0.017	0.017	0.017	0.017	0.017	0.017	0.018	0.017
2	PoAstV-2/Swine/CHI/CF2673/2017	0.533		0.021	0.020	0.020	0.018	0.025	0.017	0.017	0.018	0.018	0.018	0.018	0.018	0.018	0.019	0.019	0.018
3	PoAstV-2/Swine/CHI/FB148/2015	0.553	0.397		0.021	0.022	0.019	0.030	0.018	0.019	0.019	0.019	0.019	0.019	0.019	0.019	0.019	0.019	0.019
4	JF713710.1_Porcine_astrovirus_2_43/USA	0.539	0.298	0.399		0.021	0.017	0.025	0.017	0.018	0.018	0.018	0.018	0.018	0.018	0.018	0.019	0.019	0.018
5	LC201592.1_PoAstV2/JPN/Ishi-Ya8/2015	0.650	0.637	0.639	0.639		0.019	0.027	0.019	0.017	0.019	0.019	0.019	0.019	0.019	0.019	0.020	0.018	0.018
6	PoAstV-4/Swine/CHI/FB016/2017	0.815	0.788	0.802	0.794	0.790		0.033	0.021	0.020	0.018	0.019	0.019	0.019	0.019	0.019	0.019	0.018	0.019
7	PoAstV-4/Swine/Chile/FBO36/2017	0.795	0.840	0.811	0.830	0.790	0.448		0.031	0.034	0.029	0.029	0.029	0.028	0.028	0.028	0.029	0.028	0.027
8	JF713713.1_Porcine_astrovirus_4_35/USA	0.800	0.815	0.811	0.816	0.798	0.513	0.338		0.020	0.018	0.018	0.018	0.018	0.018	0.018	0.019	0.018	0.018
9	LC201600.1_PoAstV4/JPN/Bu4-2-2/2014	0.798	0.817	0.792	0.798	0.804	0.590	0.435	0.572		0.017	0.018	0.017	0.018	0.017	0.018	0.017	0.017	0.017
10	PoAstV-5/Swine/CHI/CF2671/2017	0.829	0.824	0.818	0.809	0.794	0.807	0.778	0.820	0.834		0.012	0.011	0.011	0.011	0.011	0.012	0.014	0.021
11	PoAstV-5/Swine/CHI/CF2672/2017	0.838	0.819	0.822	0.813	0.806	0.798	0.792	0.812	0.832	0.080		0.007	0.006	0.007	0.006	0.011	0.014	0.021
12	PoAstV-5/Swine/CHI/FB016/2017	0.831	0.813	0.815	0.806	0.804	0.802	0.792	0.812	0.827	0.077	0.028		0.006	0.006	0.006	0.012	0.014	0.021
13	PoAstV-5/Swine/CHI/FB032/2017	0.833	0.817	0.813	0.810	0.802	0.804	0.789	0.814	0.830	0.070	0.021	0.021		0.002	0.000	0.011	0.014	0.021
14	PoAstV-5/Swine/CHI/FB033/2017	0.833	0.817	0.813	0.810	0.802	0.804	0.789	0.814	0.832	0.070	0.025	0.025	0.003		0.002	0.011	0.014	0.021
15	PoAstV-5/Swine/CHI/FB036/2017	0.833	0.817	0.813	0.810	0.802	0.804	0.789	0.814	0.830	0.070	0.021	0.021	0.000	0.003		0.011	0.014	0.021
16	PoAstV-5/Swine/CHI/FB148/2015	0.836	0.810	0.818	0.799	0.799	0.806	0.799	0.820	0.845	0.098	0.082	0.085	0.073	0.076	0.073		0.015	0.022
17	JF713711.1_Porcine_astrovirus_5_33/USA	0.829	0.800	0.815	0.800	0.813	0.809	0.803	0.827	0.834	0.149	0.147	0.138	0.145	0.149	0.145	0.154		0.021
18	LC201619.1_PoAstV5/JPN/Ishi-Im1-1/2015	0.842	0.817	0.824	0.817	0.827	0.803	0.820	0.825	0.838	0.370	0.375	0.372	0.374	0.374	0.374	0.379	0.372	

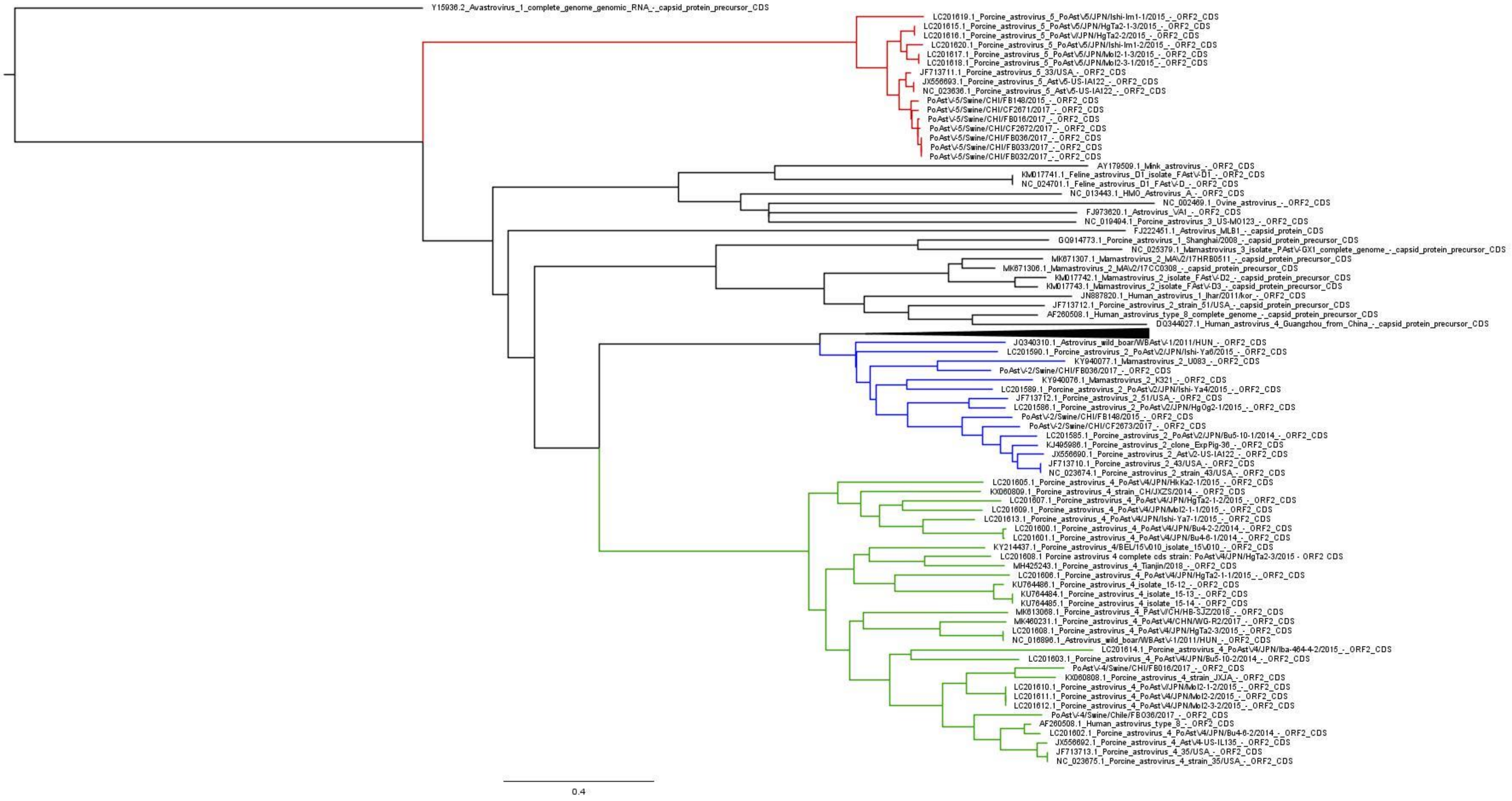

Supplementary Figure 1. Phylogenetic tree of PoAstV by using the ORF2 region. The final dataset included 93 sequences. Clusters by species are highlighted in colors: PoAstV-5 (Red), PoAstV-4 (Green) and PoAstV-2 (Blue).
